# Are both COVID-19 and COVID-19 vaccines associated with cancer?

**DOI:** 10.18632/oncotarget.28918

**Published:** 2026-09-11

**Authors:** Raphael Lataster

**Affiliations:** ^1^Independent Researcher, The University of Sydney, Sydney, NSW 2000, Australia

**Keywords:** COVID-19, COVID-19 vaccines, cancer, double standards

***Commentary on:** Kuperwasser C, El-Deiry WS. COVID vaccination and post-infection cancer signals: Evaluating patterns and potential biological mechanisms. Oncotarget. 2026; 17:1–29.* https://doi.org/10.18632/oncotarget.28824. [PubMed]

The *Oncotarget* journal has done a commendable job in the last few months of considering an alternative hypothesis: that COVID-19 vaccines, as well as COVID-19, could be contributing to cancer. An intriguing possibility given rising cancer rates [1]. Particularly interesting is the review by Kuperwasser and El-Deiry, which examines the increasing evidence and probes into the potential mechanisms at play [2]. For these efforts, the journal and editors are under attack. As part of my ongoing research into the double standards when it comes to associating COVID-19 vaccines with various outcomes, with observational research associating the vaccines with positive outcomes typically being accepted unquestionably whilst observational research associating the vaccines with negative outcomes almost always face harsh criticisms, I wish to add relevant context here by highlighting the prestigious *Nature* journal’s asymmetric standards on this topic.

A recent report in Springer Nature’s *Nature* journal discussed the possibility that COVID-19 has potentially “roused” cancer cells, that respiratory virus infections can “awaken the cells” and cause them to proliferate “to enormous numbers”. Underlying mechanisms were suggested, such as COVID-19’s ability to cause inflammation, raise IL-6 levels, and raise helper T cell levels. Furthermore, data from the UK found an “increased risk of cancer-related death in those who tested positive for COVID-19” [3]. All of this can also be said for COVID-19 vaccines, with much of the evidence published in *Nature* journals. COVID-19 vaccines are known to cause inflammation, apart from myocarditis and pericarditis, partly attributed to the spike protein [4]. They are also associated with very large increases in IL-6 levels [5]. They are also widely regarded as raising helper T cell levels [6].

Additionally, while COVID-19 has been associated with cancer, the supplementary material of an Australian study on the mortality benefits of COVID-19 vaccination indicated that COVID-19 vaccination may also be associated with cancer [7]. Furthermore, another study has noted that the m1Ψ modification of mRNA COVID-19 vaccines “stimulated cancer growth and metastasis… suggesting that COVID-19 mRNA vaccines could aid cancer development” [8]. The *Nature* report does not note these associations, however, and even suggests that cancer survivors should “consider vaccination against pathogens such as SARS-CoV-2” until more is known.

I did alert the journal to these mechanistic links also existing between cancer and the vaccines, but the editors were uninterested. Given this evidence, however, published in highly regarded scientific journals, if we are to consider if COVID-19 is contributing to cancers, should we not also consider if COVID-19 vaccines are contributing to cancers? Such inquiries should proceed without preconceptions, applying the same evidentiary standards regardless of whether findings implicate infection, vaccination, or both. The willingness of *Oncotarget* to host this discussion is a welcome step toward open scientific dialogue on these complex intersections.
